# Arthroscopic removal of loose bodies in synovial chondromatosis of shoulder joint, unusual location of rare disease: A case report and literature review

**DOI:** 10.1016/j.amsu.2018.11.016

**Published:** 2018-12-05

**Authors:** Hussain Wahab, Obada Hasan, Ahmed Habib, Naveed Baloch

**Affiliations:** Department of Orthopaedics, Aga Khan University Hospital AKUH, Karachi, Pakistan

**Keywords:** Arthroscopic synovectomy, Shoulder arthroscopy, Synovial chondromatosis, Loose bodies

## Abstract

**Introduction:**

Synovial chondromatosis is a benign mono-articular arthropathy affecting synovial joints. It mostly affects knee joint, followed by hip, elbow and wrist and is rarely reported for shoulder joint. The exact pathogenesis is not known. Usual symptoms are pain, difficulty in movement due to mechanical obstruction. The classic treatment is arthrotomy, removal of chondromatoid loose bodies and synovectomy. With recent advances arthroscopic removal of the chondromatoid loose bodies is a good option with relatively better post op rehabilitation and faster recovery.

**Case presentation:**

20 years old gentleman presented to clinic with history of pain in right shoulder for 2 years and decreased range of motion. There was no history of trauma or fever. Work up done and diagnosed with synovial chondromatosis. Arthroscopic removal of chondromatoid loose bodies and synovectomy was done. More than 120 loose bodies were removed. On two (2) years follow-up patient is pain free and having full range of motion at right shoulder joint.

**Discussion:**

Synovial chondromatosis is rare in shoulder joint. The Primary synovial chondromatosis of unknown etiology, and secondary synovial chondromatosis due to degenerative joint disease. Classic treatment is arthrotomy and synovectomy. With recent advances, arthroscopic removal of loose bodies and synovectomy is also a good option for its treatment. In literature only few cases have been reported treated with arthroscopic removal of loose bodies and synovectomy.

**Conclusion:**

Arthroscopic treatment of synovial chondromatosis is a good option if expertise is available. It causes less surgical trauma, better visualization during surgery, early recovery.

## Highlights

• Patient had history of recurrent pain, relieved on analgesics.
• No intra articular injection was given before surgery.
• Procedure was performed by an orthopedic surgeon with super-specialty in sports medicine. Post rehabilitaion started on first post op day, on follow up vistis patient was asymptomatic.

## 1 Introduction

Synovial chondromatosis is a benign idiopathic disease affecting lining of articular surfaces (synovium) of synovial joints. It is a rare condition causing unusual mono-articular pathology. Worldwide male to female ratio is 2:1, and the common age group affected is 20–40 years [1]. The exact pathogenesis is not known but it is suggested that synovium undergoes cartilage metaplasia. The synovium grows abnormally and produces small nodules of cartilage which may ossify or not. The synovial chondroid-metaplastic focus becomes pedunculated and then by breaking off, becomes a loose body within the joint [2].

It affects large joints mostly Knee (70%) followed by Hip (20%), elbow, wrist, ankle and least commonly shoulder joint [3]. Symptoms are usually non-specific. Restrictions in joint movements due to mechanical effects of loose bodies, pain and swellings are observed during periods of active use. Recurrence is common (up to 31%. [2]) and in pre-operative counseling it should be explained to the patient. The classic treatment of synovial chondromatosis is arthrotomy and removal of loose bodies and affected synovium. There is scarcity of the relevant literature about this very rare condition and more paucity about the arthroscopic management of such conditions. Available data was in the form of case reports and small case series only [[4], [5], [6], [7], [8], [9]].

This case report is about a patient with primary synovial chondromatosis who underwent arthroscopic removal of more than 120 synovial chondromatoid loose bodies and partial synovectomy of shoulder joint which is unusual location of this rare disease with 2 years follow-up.

The work has been reported in line with the SCARE criteria [25]. In author's knowledge there is only 1 case published which reports the arthroscopic removal of more than 100 chondromatoid bodies from shoulder joint in literature.

## 2 Case report

**Fig. 1:**
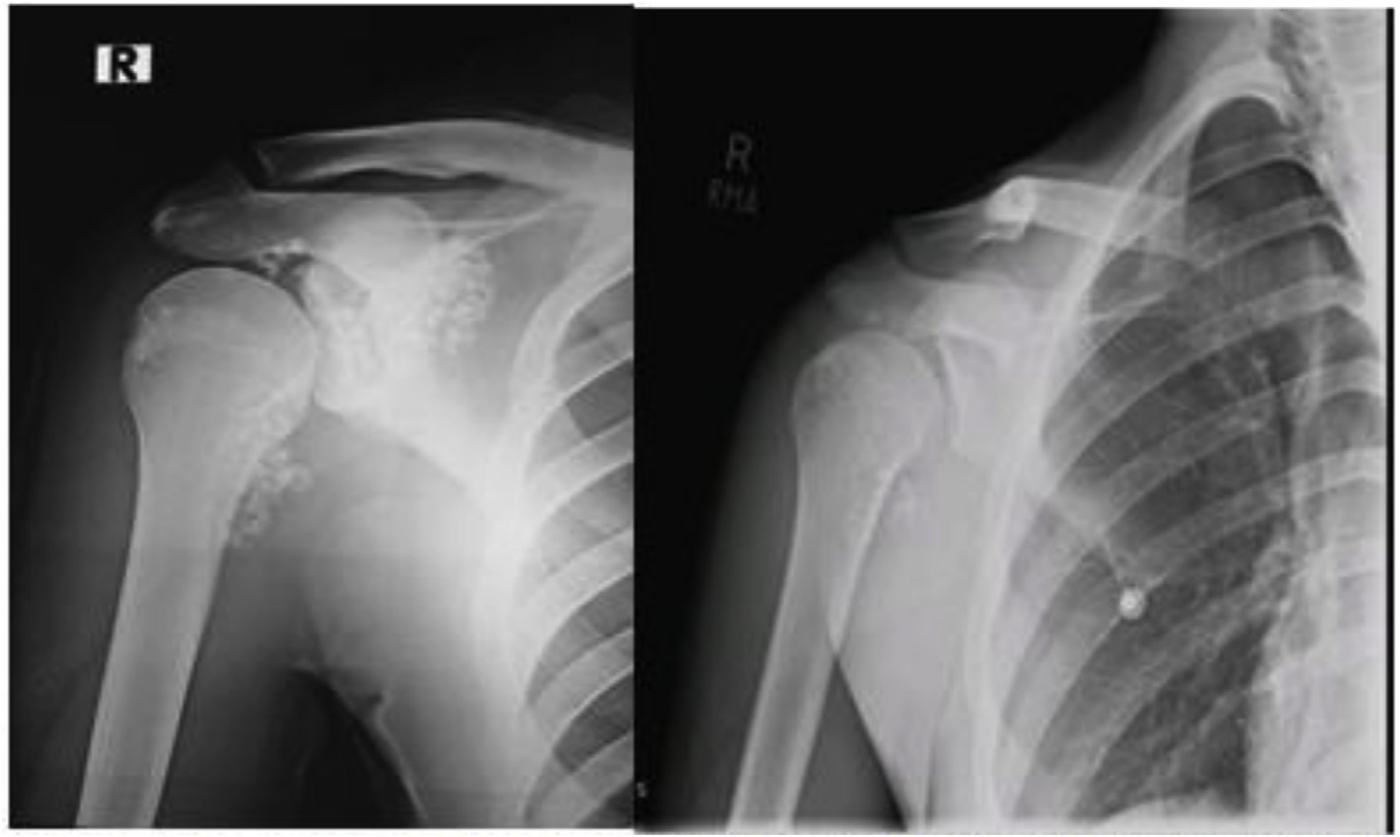
On left side pre operative x-ray showing the extensive loose bodies in shoulder joint on right side postoperative x-ray after removal of the loose bodies.

**Fig. 2:**
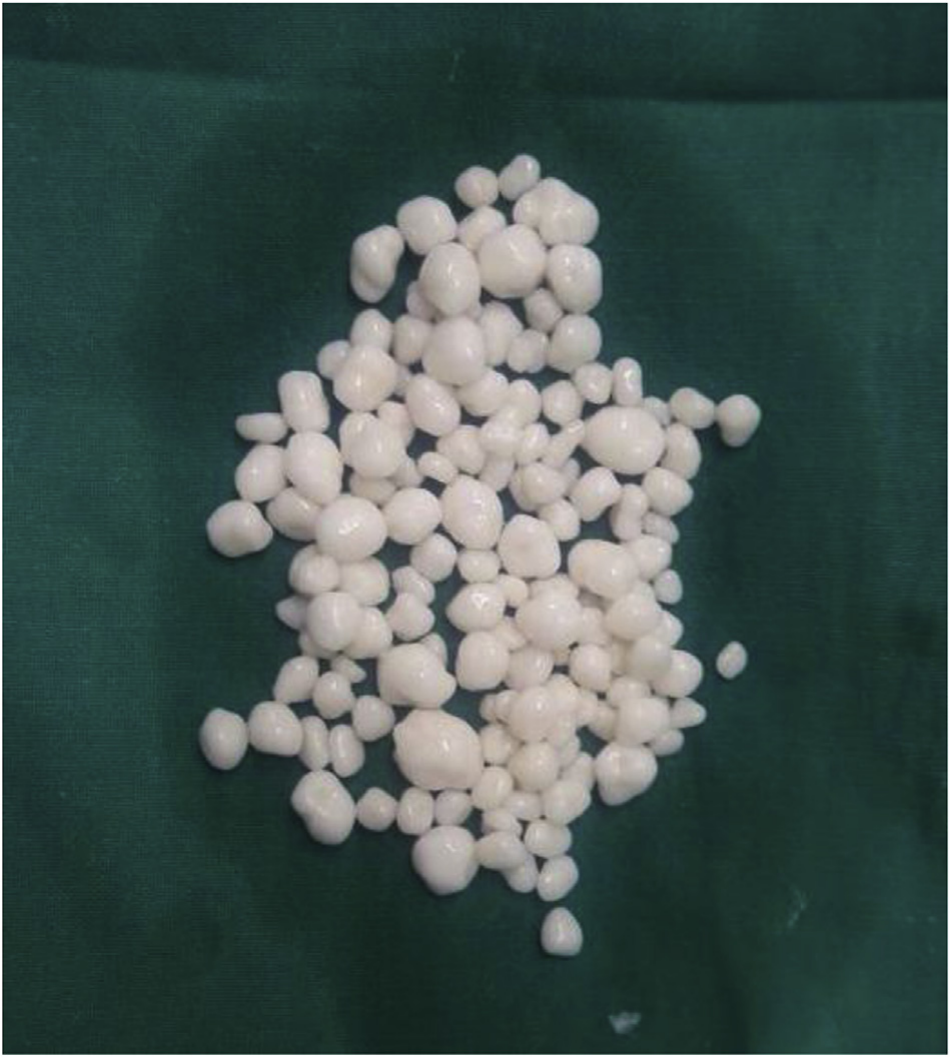
Pearly white loose bodies of different sizes.

**Fig. 3:**
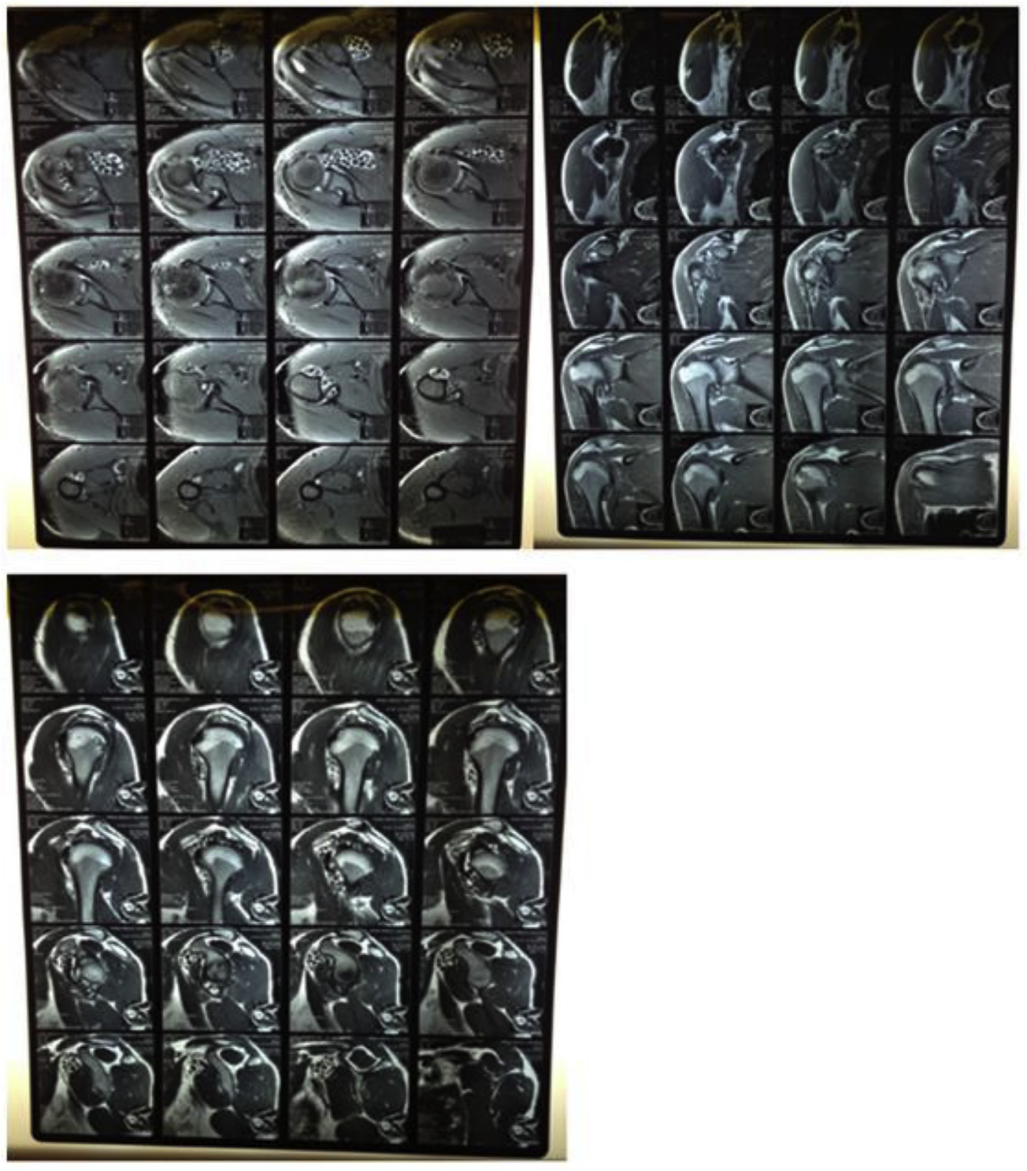
Pre operative MRI was done to know exact location of loose bodies and to look for other pathologies they are loose bodies and synovitis no other pathology or tendenopathy was identified upper left image axial view of shoulder joint showing loose bodies upper right image coronal view of shoulder joint showing loose bodies pre operative MRI sagittal view of shoulder joint showing loose bodies.

A 20 years old male presented with history of intermittent right shoulder pain, for last 2 years. There was no history of trauma or fever. On examination he was a young gentleman with average height and built with no obvious deformity of the shoulder joint, range of motion was normal although extremes of movements at right shoulder were painful. Plain radiographs that included shoulder antero-posterior and scapular Y-views showed radio opaque densities in right gleno-humeral cavity, sub acromial space and medial aspect of proximal humerus (Fig. 1, Left side). Provisional diagnosis of synovial chondromatosis was made. His MRI showed presence of multiple chondromatoid bodies and no other pathology. He was planned for arthroscopic removal of loose bodies. Arthroscopy was performed which revealed extensive synovitis and multiple loose chondromatoid bodies. All loose bodies were removed including those which were still attached to synovium but clearly visible or palpable. Post-Operative x-rays shows clearance of most of the loose bodies (Fig. 1 Right side). Clinical picture of the chondromatoid bodies is shown in Fig. 2. Axial, sagittal and coronal images of MRI are shown in Fig. 3 …

Post Op full range of motion was allowed. At 2 years follow-up, there were no symptoms and range of motion was normal.

## 3 Discussion

Synovial chondromatosis is rare disease. Two forms of the disease explained. Primary synovial chondromatosis of unknown etiology also called Reichel syndrome, and secondary synovial chondromatosis due to degenerative joint disease, such as osteoarthritis, rheumatoid arthritis, osteonecrosis neuropathic osteo-arthropathy [10]. Three stages for the disease process explained [1]: active disease without intra-articular loose bodies [2], transitional lesions with synovial proliferation and loose bodies, and [3] loose bodies without synovial disease [8]. In this case report, we assumed this as a primary synovial chondromatosis due to the observation of almost the same ovoid shaped look of loose bodies in the arthroscopy, with no underlying joint disorders and with normal blood parameters.

Most common sites involved include the knee and hip joints [11,12]. Other joints rarely reported to be involved include the distal radio ulnar joint, the acromioclavicular joint [13], the facet joint [14], the temporo-mandibular joint, the metacarpo-phalangeal joint [15], and the least common gleno-humeral joint [16]. In our case it occurred in the least often site, the shoulder joint. Although the term “SNOW STORM KNEE” has been used to describe this condition in the knee joint as is observed by arthroscopy, which presents as white ossified loose bodies on the synovial membrane of the involved joint [8]. This appearance was also noted in our case.

These lose bodies can cause locking of the joints, damage to articular surface, irritation of tendons causing tendinitis and, although rare, transformation to chondrosarcoma [17]. There is no uniform radio graphical or MR imaging appearance for synovial chondromatosis. It may not be detected in 5–30% of the cases if the loose bodies lack the calcification [18].

## 4 Treatment options include

The treatment of either primary or secondary synovial chondromatosis requires surgery, which involves the removal of the loose bodies. The standard method for treatment of the shoulder has been open arthrotomy and synovectomy [19]. The recommended treatment is the combined removal of the loose bodies with synovectomy due to the lower recurrent rates by the use of this method [20].

The advantages of arthroscopic removal are; good visualization during surgery, low morbidity, less surgical trauma, early recovery and rehabilitation. Pre-operative counseling should include the recurrence rate as well as the regular follow-up needed.

Sports medicine and shoulder arthroscopy particularly is an underdeveloped field in our region and very few centers in Pakistan offer the facility with our institution being the pioneer in starting shoulder arthroscopy. Due to lack of equipment we were too restricted to diagnostic shoulder arthroscopy and subacromial decompression mainly until induction of a fellowship trained surgeon in sports medicine and arthroscopic surgery. This patient presented and worked up in a peripheral hospital and was referred to our hospital with a provisional diagnosis and in a hope to avoid open surgery. At that time all shoulder arthroscopic inventory was still not available in Pakistan including cannulas to maintain arthroscopy portals.

Only feasible idea to remove such a large quantity of loose bodies was to irrigate the joint and let the majority of loose bodies come through the portals kept open via large cannulas. Handpicking individual loose bodies with instruments would have been a trying job. Lack of availability of cannulas gave us innovative idea of converting 3 cc BP empty syringe in a cannula to keep the port open and hence washing most of loose bodies with irrigating fluid. Remaining loose bodies in corners and those still attached to synovium were picked with a grasper.

Limited synovectomy was performed with ablator in areas where loose bodies were visible or palpable.

**Table:** Literature review:

Murphy et al [7]	1910–1957	Out of 32 cases only 1 shoulder patient
Mil-gram [8]		4 out of 31 case involving shoulder
Imhoff and Schreiber [9]	1957–1987	Out of 33 cases, no shoulder cases
Dorfmann H et al. [21]	1989	Arthroscopic removal of synovial chondromatosis of knee in 39 cases
Ferro FP et al. [22]	2015	Arthroscopic treatment of hip synovial chondromatosis
Urbach D et al. [19]	2008	5 cases of shoulder arthroscopic removal of chondromatoid loose bodies and poartial synovectomy with 4–9 years follow up
Antonio Jiménez-Martín et Al [23]	2014	Arthroscopic removal of 9 loose bodies from shoulder joint, with 2 years follow up
TahirMutluDuymus et Al [2]	2015	Arthroscopic removal of 33 loose bodies from shoulder joint, with 1 year follow up
PradyumnaRaval et al. [24]	2016	Arthroscopic removal of 126 loose bodies from left shoulder with 3 months follow up

The largest series in the literature on the treatment of synovial chondromatosis are by Murphy et al [7] Mil-gram [8], and Imhoff and Schreiber [9]. The majority of their 95 cases concerned the knee, hip, and elbow. Murphy found only 1 shoulder patient out of 32patients with synovial chondromatosis between 1910 and 1957. Milgram in his multi institutional study documented 4 out of 31 cases involving the shoulder. Imhoff and Schreiber, reporting on 33 cases occurring between 1957 and 1987, had no cases involving the shoulder. The clinical results of open arthrotomy and synovectomy in these cases were described as good.

Dorfmann H et al. reported arthroscopic removal of the synovial chondramatoid of knee joint in 39 patients. 3.5 years follow of 29 patients was done. In 78% cases good results were obtained [21].

Ferro FP et al., conducted study on outcomes of arthroscopic treatment of hip synovial chondromatosis. With a 2.5 years follow up they concluded that arthroscopic removal of chondramatoid bodies in hip joint synovial chondromatosis with aggressive rehabilitation was effective in relieving symptoms associated with synovial chondromatosis [22].

Urbach D et al., reviewed 5 cases of shoulder arthroscopic removal of chondromatoids and partial synovectomy. These patients were followed for 4–9 years. Outcome was very good, in 2 out of 5 cases there was recurrence or persistent chondromatosis on radiology. No revision surgery was required in any case [19].

Antonio Jiménez-Martín et Al reported, 9 loose bodies were arthroscopically removed from shoulder joint in a 53 years old gentleman with a 2 years follow up [23].

TahirMutluDuymus et Al reported, 33 loose bodies removed from right shoulder of a 33 years old female, with a follow up of 1 year [2].

PradyumnaRaval et al. reported removal of 126 loose bodies from left shoulder of a 52 years old gentleman, with 3 months follow up [24].

In our case we extracted more than 120 loose bodies from right shoulder through arthroscopy, those which were deep or adherent to synovium left in place. On 2 years follow up, patient was pain free with full range of motion of his shoulder and there was no recurrence of symptoms.

## 5 Conclusion

Arthroscopic treatment of synovial chondromatosis is a good option if expertise is available. It causes less surgical trauma, better visualization during surgery, early recovery and rehabilitation. Patient should be informed about recurrence rate before surgery.

## Consent

This is to confirm that the case report, titled Arthroscopic removal of loose bodies in Synovial Chond romatosis of Shoulder joint, unusual location of rare disease: A case report and literature review, was approved by our university hospital ethical review committee (ERC) with file no. 4985 25-Sep- 17. The Associate Professor, & Section Head Orthopaedics takes responsibility that exhaustive attempts have been made to contact the patient. Furthermore the paper has been sufficiently anonymised not to cause harm to the patient or their family and thus allowed for publications.

## Provenance and peer review

Not commissioned externally peer reviewed.

## Ethical approval

Research ethics approval was obtained by the hospital Ethical Review Committee. ERC number 4985 25-Sep-17.

## Sources of funding

Nil.

## Author contribution

Study design: Hussain Wahab.

Data collection: Hussain Wahab.

Statistical analysis: NA.

Writing first draft of manuscript: Dr Obada, Dr Ahmed, Dr Naveed, Dr Hussain.

Critical review and approval of manuscript: All.

## Conflicts of interest

No conflicts to declare.

## Research registration number

UIN: researchregistry3194.

## Guarantor

Hussain Wahab.
