# Supplementary material for: Arthroscopic removal of loose bodies in synovial chondromatosis of shoulder joint, unusual location of rare disease: A case report and literature review
Source: Ann Med Surg (Lond). 2018 Dec 5;37:25–9. doi: 10.1016/j.amsu.2018.11.016 (PMC6297053; doi:10.1016/j.amsu.2018.11.016)
Supplement: Multimedia component 1 [file mmc1.docx]

| **SCARE Checklist** | | | |
| --- | --- | --- | --- |
| **Topic** | **Item** | **Checklist item description** | **Page Number** |
| **Title** | **1** | **Arthroscopic removal of loose bodies in Synovial Chondromatosis of Shoulder joint, unusual location of rare disease: A case report and literature review** |  |
| **Key Words** | **2** | Synovial chondromatosis, Shoulder arthroscopy, loose bodies. |  |
| **Abstract** | **3a** | Synovial chondromatosis is a benign mono articular arthropathy affecting articular surface of joint. It mostly affects knee joint, followed by hip, elbow and wrist. The exact pathogenesis is not known. Usual symptoms are pain, difficulty in movement due to mechanical obstruction of the loose bodies. The classic treatment is arthrotomy and removal of chondromatoid loose bodies and synovectomy. With recent advances arthroscopic removal of the chondramatoid loose bodies is a good option with relatively better post op rehabilitation and faster recovery time |  |
|  | **3b** | 20 years old gentleman presented to our clinic history of pain right shoulder for 2 years and decreased range of motion. There was no history of trauma or fever. |  |
|  | **3c** | Work up done and diagnosed with synovial chondromatosis. Arthroscopic removal of chondromatoid loose bodies and synovectomy was done. More than 120 loose bodies were removed. On 1.5 years follow up patient is pain free and having full range of motion at right shoulder joint. Classic treatment is arthrotomy and synovectomy. With recent advances, arthroscopic removal of loose bodies and synovectomy is also a good option for its treatment carrying many advantages over the classic open method. In literature only few cases have been reported treated with arthroscopic removal of loose bodies and synovectomy. |  |
|  | **3d** | Arthroscopic removal of loose bodies in synovial chondromatosis from shoulder is a good option. It causes less surgical trauma and better post op rehabilitation |  |
| **Introduction** | **4** | Synovial chondromatosis is a benign idiopathic disease affecting lining of articular surfaces (synovium). It is a rare condition causing unusual mono-articular pathology. Worldwide the exact prevalence is not known. Male to female ratio is 2:1, and the common age group affected is 20 to 40 years (1 ). The exact pathogenesis is not known but it is suggested that synovium undergoes cartilage metaplasia. The synovium grows abnormally and produces small nodules of cartilage which may ossify or not. The synovial chondroidmetaplastic focus becomes pedunculated and then by breaking off, becomes a loose body within the joint.  It mostly affects large joints, Knee (70%), followed by Hip (20%), elbow, wrist ankle and least common shoulder joint. Symptoms are usually non-specific. Restrictions in joint movements due to mechanical effects of loose bodies, pain and swellings are observed during periods of active use. Recurrence is common and in per operative counseling it should be explained to the patient. Recurrence rate reported up to 31 %.  The classic treatment of synovial chondromatosis is arthrotomy and removal of loose bodies and affected synovium. There is scarcity of the relevant literature about this very rare condition and more paucity about the arthroscopic management of such conditions. Available data was in the form of case reports and small case series only.This case report is about a patient with primary synovial chondromatosis who underwent arthroscopic removal of more than 120 synovial chondramatoid loose bodies and partial synovectomy of shoulder joint which is unusual location of this rare disease with 1 year and 6 months follow-up. |  |
| **Patient Information** | **5a** | 20 years old boy, resident of Mardan (Pakistan), student by profession. Right hand dominant. |  |
|  | **5b** | Presented with history of intermittent right shoulder pain, for last 2 years. There was no history of trauma or fever. |  |
|  | **5c** | Past medical history un-remarkable. |  |
|  | **5d** | Patient has history analgesics use for pain relief, otherwise past history is un-remarkable |  |
| **Clinical Findings** | **6** | On examination he was a young gentleman with average height and built with no obvious deformity of the shoulder joint, range of motion was normal although extremes of movements at right shoulder were painful. |  |
| **Timeline** | **7** |  |  |
| **Diagnostic Assessment** | **8a** | Plain radiographs that included shoulder antero-posterior and scapular Y-views showed radio opaque densities in right gleno-humeral cavity, sub acromial space and medial aspect of proximal humerus. (Figure 1) provisional diagnosis of synovial chondromatosis was made and MRI of right shoulder was also done to rule out other concurrent pathology. MRI showed presence of multiple chondroid bodies and no other pathology. He was planned for arthroscopic removal. |  |
|  | **8b** |  |  |
|  | **8c** |  |  |
|  | **8d** |  |  |
| **Therapeutic Intervention** | **9a** | Arthroscopy was performed which revealed extensive synovitis and multiple loose chondramatoid bodies. Partial synovectomy done and loose bodies were removed including those which were still attached to synovium but clearly visible or palpable. Post-Operative x-rays shows clearance of most of the loose bodies. Post Op full range of motion was allowed. |  |
|  | **9b** |  |  |
|  | **9c** |  |  |
|  | **9d** | Orthopaedics surgeon, who has super speciality in sports medicine. |  |
|  | **9e** |  |  |
|  | **9f** | Post op rehabilitation was started at Aga khan university hospital. Range of motion exercises were started on 1^st^ post Operative day. |  |
| **Follow-up and**  **Outcomes** | **10a** | At 2 years follow-up, there were no symptoms and range of motion was normal. |  |
|  | **10b** |  |  |
|  | **10c** | Surgery done at Aga khan university hospital, post op rehabilitation protocol was followed and patient was compliant with rehabilitation protocol |  |
|  | **10d** |  |  |
| **Discussion** | **11a** | Synovial chondromatosis is rare disease. Two forms of the disease explained. Primary synovial chondromatosis of unknown etiology also called Reichel syndrome, and secondary synovial chondromatosis due to degenerative joint disease, such as osteoarthritis, rheumatoid arthritis, osteonecrosis neuropathic osteoarthropathy. Three stages for the disease process explained: active disease without intraarticular loose bodies, transitional lesions with synovial proliferation and loose bodies, and loose bodies without synovial disease.  In this case report, we assumed this as a primary synovial chondromatosis due to the observation of almost the same ovoid shaped look of loose bodies in the arthroscopy, with no underlying joint disorders and with normal blood parameters.  Most common sites involved include the knee and hip joints. Other joints reported rarely to be involved include the distal radioulnar joint, the acromioclavicular joint, the facet joint, the temporomandibular joint, the metacarpophalangeal joint, and the least common glenohumeral joint . In our case it occurred in the least often site, the shoulder joint. Although the term “SNOW STORM KNEE” has been used to describe this condition in the knee joint as is observed by arthroscopy, which presents as white ossified loose bodies on the synovial membrane of the involved joint. This appearance was also noted in our case.  These lose bodies can cause locking of the joints, damage to articular surface, irritation of tendons causing tendinitis and , although rare, transformation to chondrosarcoma. There is no uniform radiographical or MR imaging appearance for synovial chondromatosis. It may not be detected in 5-30% of the cases if the loose bodies lack the calcification.  Treatment options include:  The treatment of either primary or secondary synovial chondromatosis requires surgery, which involves the removal of the loose bodies. The standard method for treatment of the shoulder has been open arthrotomy and synovectomy. The recommended treatment is the combined removal of the loose bodies with synovectomy due to the lower recurrent rates by the use of this method.  Arthroscopic removal of synovial chondromatosis is available in our setup. The advantages of arthroscopic removal are; good visualization during surgery, low morbidity, less surgical trauma, early recovery and rehabilitation.  Pre-operative counseling should include the recurrence rate as well as the regular follow-up needed.  The largest series in the literature on the treatment of synovial chondromatosis are by Murphy et al Mil-gram, and Imhoff and Schreiber. The majority of their 95 cases concerned the knee, hip, and elbow. Murphy found only 1 shoulder patient out of 32 patients with synovial chondromatosis between 1910 and 1957. Milgram in his multi institutional study documented 4 out of 31 cases involving the shoulder. Imhoff and Schreiber, reporting on 33 cases occurring between 1957 and 1987, had no cases involving the shoulder. The clinical results of open arthrotomy and synovectomy in these cases were de-scribed as good.  [Dorfmann H](https://www.ncbi.nlm.nih.gov/pubmed/?term=Dorfmann%20H%5BAuthor%5D&cauthor=true&cauthor_uid=2706051) et al reported arthroscopic removal of the synovial chondramatoid of knee joint in 39 patients. 3.5 years follow of of 29 patients was done. In 78 % cases good results were obtained .  Ferro FP et al, conducted study on outcomes of arthroscopic treatment of hip synovial chondromatosis. With a 2.5 years follow up they concluded that arthroscopic removal of chondramatoid bodies in hip joint synovial chondromatosis with aggressive rehabilitation was effective in relieving symptoms associated with synovial chondromatosis.  Urbach D et al, reviewed 5 cases of shoulder arthroscopic removal of chondromatoids and partial synovectomy. These patients were followed for 4 to 9 years. Outcome was very good, in 2 out of 5 cases there was recurrence or persistent chondromatosis on radiology. No revision surgery was required in any case.  Antonio Jiménez-Martín et Al reported, 9 loose bodies were arthroscopically removed from shoulder joint in a 53 years old gentleman with a 2 years follow up.  TahirMutluDuymus et Al reported , 33 loose bodies removed from right shoulder of a 33 years old female, with a follow up of 1 year.  PradyumnaRaval et al reported removal of 126 loose bodies from left shoulder of a 52 years old gentleman, with 3 months follow up.  In our case we extracted more than 120 loose bodies from right shoulder through arthroscopy, those which were deep or adherent to synovium left in place. On 1 year and 6 months follow up, patient was pain free with full range of motion of his shoulder and there was no recurrence of symptoms. |  |
|  | **11b** |  |  |
|  | **11c** |  |  |
|  | **11d** |  |  |
| **Patient Perspective** | **12** | Started daily activities, patient is asymptomatic and satisfied with the treatment |  |
| **Informed Consent** | **13** | Yes. |  |
| **Additional 1Information** | **14** | NO Conflicts of Interest, no sources of funding, institutional review board or ethical committee approval obtained. |  |
